# DNA Barcoding for the Identification of Adult Mosquitoes (Diptera: Culicidae) in Western Australia

**DOI:** 10.1002/ece3.70493

**Published:** 2024-11-08

**Authors:** Binit Lamichhane, Craig Brockway, Kimberly Evasco, Jay Nicholson, Peter J. Neville, John S. Mackenzie, David Smith, Allison Imrie

**Affiliations:** ^1^ School of Biomedical Sciences University of Western Australia Perth Western Australia Australia; ^2^ Environmental Health Directorate Department of Health Perth Western Australia Australia; ^3^ Faculty of Health Sciences Curtin University Bentley Western Australia Australia; ^4^ PathWest Laboratory Medicine Perth Western Australia Australia

**Keywords:** Culicidae, DNA barcoding, mitochondrial COI, mosquitoes, Western Australia

## Abstract

Precise mosquito identification is integral to effective arbovirus surveillance. Nonetheless, the conventional morphological approach to identifying mosquito species is laborious, demands expertise and presents challenges when specimens are damaged. DNA barcoding offers a promising alternative, surmounting challenges inherent in morphological identification. To integrate DNA barcoding into arbovirus surveillance effectively, a robust dataset of mosquito barcode sequences is required. This study established a comprehensive repository of Cytochrome Oxidase I (COI) barcodes, encompassing 177 samples representing 45 mosquito species from southern and northern Western Australia (WA), including 16 species which have not been previously barcoded. The average intraspecific and interspecific genetic distances were 1% and 6.8%, respectively. *Anopheles annulipes* sensu lato had the highest intraspecific distance at 9.1%, signifying a genetically diverse species. While validating the potential of COI barcodes to accurately differentiate mosquito species, we identified that some species pairs have low COI divergence. This includes *Aedes clelandi* and *Ae. hesperonotius*, *Tripteroides atripes* and *Tp. punctolaeralis* and *Ae. turneri* and *Ae. stricklandi*. In addition, we observed ambiguity in identification of the members of *Culex sitiens* subgroup (*Cx. annulirostris*, *Cx. palpalis* and *Cx. sitiens*) and three members of Cx. pipiens complex (*Cx. australicus*, *Cx. globocoxitus*, *Cx. quinquefasciatus*). In summary, despite presenting challenges in the identification of some mosquito species, the COI barcode accurately identified most of the species and generated a valuable resource that will support the WA arbovirus surveillance program and enhance public health intervention strategies for mosquito‐borne disease control.

## Graphical abstract

DNA barcodes of 45 species of Western Australian mosquitoes sampled across a wide spatial range, were obtained using the universal COI barcode. Most species exhibited adequate genetic diversity, enabling reliable species identification. The barcodes generated in this study can serve as valuable resources for mosquito surveillance programs, aiding in species identification, enhancing our understanding of evolutionary relationships and identifying patterns of associations among different mosquito species.

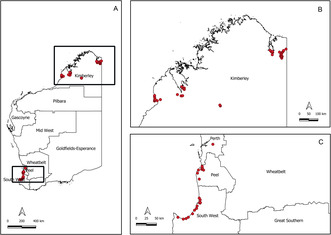

## 1 Introduction

Accurate mosquito identification is of utmost importance for effective arbovirus surveillance, serving as a crucial prerequisite for the successful control of vector‐borne diseases. Traditional methods of field‐collected mosquito identification primarily rely on Linnaean taxonomic approaches, centred around the meticulous observation of morphological characteristics. These methods, often reliant on dichotomous keys, are notorious for their time‐intensive nature and demanding years of specialised training for reliable application. Moreover, the accuracy of morphological identification hinges on the preservation of sample integrity, a criterion that is not consistently attainable in the field. The potential for damage during collection introduces the possibility of error. In response to these challenges, DNA barcoding has emerged as a promising alternative that may mitigate the problems associated with morphological identification (Beebe 2018). This enables us to identify mosquitoes to species and subspecies levels, understand genetic diversity and make predictions on evolution and phylogenetic relationships.

Herbert and colleagues (Hebert et al. 2003) first applied the DNA barcoding approach in 2003 and it has since proven its utility in the identification of various animal species, including mosquitoes (Beebe 2018). DNA barcoding involves the genetic identification of mosquitoes through specific gene sequences that exhibit inter‐species variability while remaining conserved within species (Hebert and Gregory 2005). The most widely adopted barcode region for animals is the 648 bp Cytochrome Oxidase I (COI) gene fragment amplified using the primer pair LCO1490 and HCO2198, often referred to as ‘universal barcode’ (Folmer et al. 1994). Other regions within the COI gene have been assessed as alternative barcodes (Endersby et al. 2013; Lunt et al. 1996). The mitochondrial COI gene is ideal for its high copy number and substantial inter‐species sequence variation (Beebe 2018). Additionally, various other genetic markers, such as internal transcribed spacer subunit 2 (ITS2), acetylcholinesterase 2 (ace‐2), elongation factor‐1 alpha, alpha amylase, NADH dehydrogenase and zinc finger, have been employed in mosquito barcoding studies (Endersby et al. 2013; Foley et al. 2007; Hasan et al. 2009; Hemmerter, Slapeta, and Beebe 2009; Puslednik, Russell, and Ballard 2012). Combining two or more of these barcodes has proven effective in distinguishing members of species complexes and subgroups, which is often challenging when using a single barcode region (Bourke et al. 2013; Foster et al. 2013). Consequently, DNA barcoding has allowed identification of less familiar species that were prone to misidentification.

In Australia, over 300 mosquito species are known to be extant and about 100 species are found in Western Australia (WA). WA, the country's largest state, covers roughly one‐third of the entire landmass. The majority of the WA population is concentrated in the southwest, while the remainder of the state remains sparsely populated. Southwest WA is deemed a high‐risk region for Ross River virus (RRV) and Barmah Forest virus (BFV) disease outbreaks, whereas mosquitoes in the northern regions of WA transmit RRV, BFV, Murray Valley Encephalitis virus (MVEV) and West Nile virus Kunjin subtype (WNV_KUN_). Within the framework of arbovirus surveillance programs, mosquitoes are routinely collected from the southwest and from northern WA specifically in the Pilbara and Kimberley regions (Cheryl Johansen et al. 2014). Currently, these mosquito specimens are identified using morphological methods and dichotomous keys. Barcoding data of WA mosquitoes is limited to only a handful of species. The COI barcode data of only six WA mosquito species are available in the Barcode of Life Database (BOLD), a cloud‐based platform for storage and curation of DNA barcodes (accessed 11/01/2024) (Ratnasingham and Hebert 2007).

This study aimed to assess the utility of DNA barcoding in identifying mosquitoes and to generate a DNA barcode library for the common mosquito species found in WA. We applied DNA barcoding to specimens from 45 species belonging to nine genera of mosquitoes collected in the southwestern and Kimberley regions of WA, each characterised by distinct climatic conditions—a temperate climate in the southwest and a tropical monsoon climate in the Kimberley. Our primary objective is to significantly expand the COI barcode database of Australian mosquito species by creating a comprehensive barcode library of mosquito species from WA. This resource will significantly enhance WA mosquito surveillance programs.

## 2 Materials and Methods

### 2.1 Mosquito Collection and Morphological Identification

**FIGURE 1:**
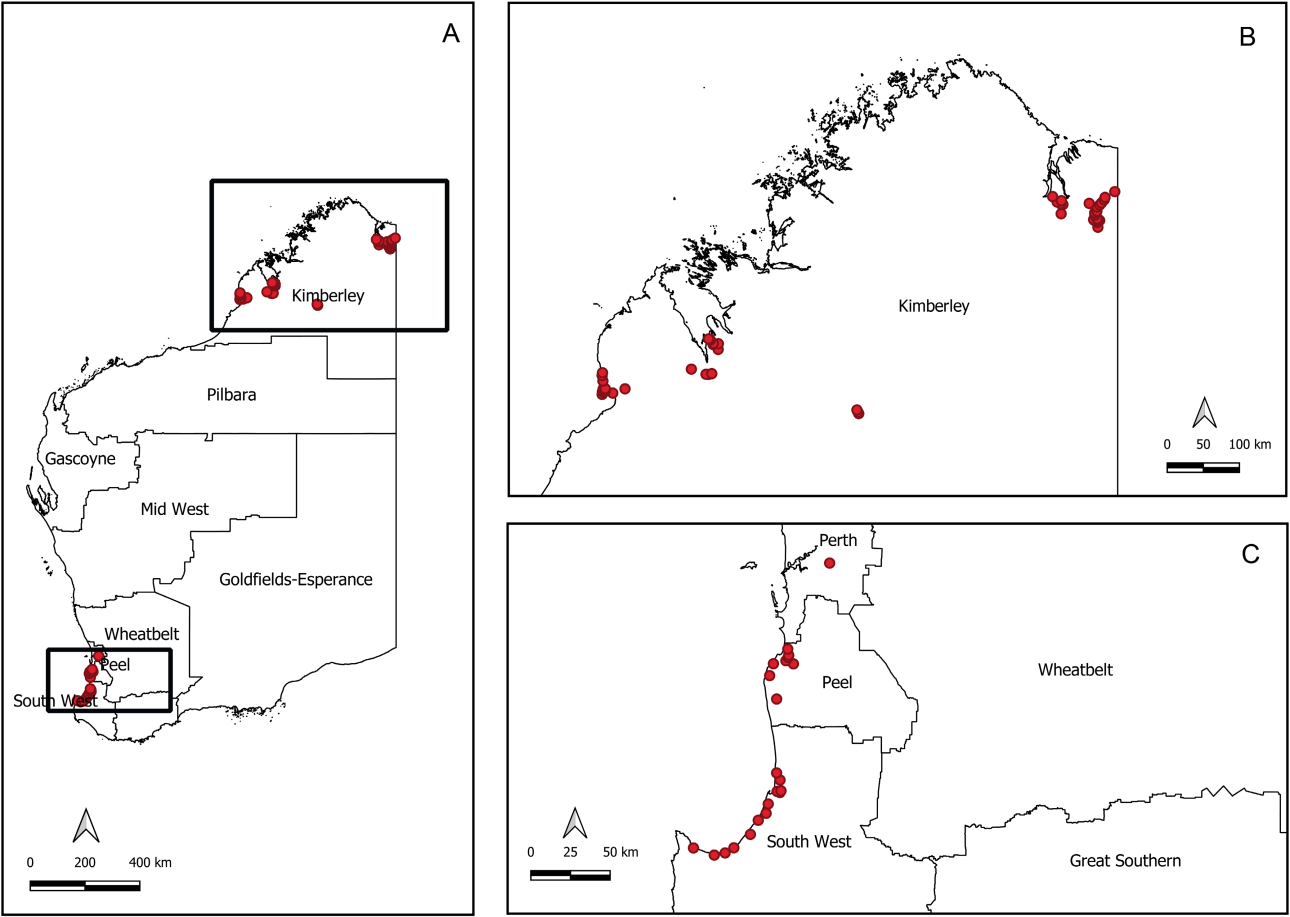
Western Australian mosquito sample locations. Mosquitoes were sampled from geographically distinct regions in Western Australia (A, boxed), including the Kimberley region (B) and sites within Perth Metropolitan, Peel and South West regions (C). Within each region, samples were collected from multiple traps sites (red dots). Maps created with QGIS 3.14.

Adult mosquitoes were collected by the Medical Entomology team of the Department of Health, WA between 2018 and 2020 as a part of the routine arbovirus surveillance program. The samples were collected from 77 different locations (Figure 1) encompassing 13 sites within the Shire of Broome, 14 in the Shire of Derby‐West Kimberley, 28 within the Shire of Wyndham East Kimberley, 21 across Peel and South West WA and 1 from the Perth metropolitan area. Mosquitoes were collected using EVS CO_2_ traps (Rohe and Fall 1979) baited with dry ice and set at each location for approximately 12 h. The captured mosquitoes were euthanized immediately upon trap collection by placing them on dry ice, transferred to labelled vials and transported to the Department of Health Medical Entomology laboratory in Perth. Upon arrival, samples were stored at −80°C until further processing.

Morphological identification of the mosquitoes was performed on refrigerated tables under stereoscopic microscopes. The species were designated based on published taxonomic keys and descriptions (Lee et al. 1984; Liehne 1991; Russell and Debenham 1996). A total of 177 mosquito specimens, representing nine genera and 45 distinct species, were included in this study (Table S1).

### 2.2 DNA Extraction, Polymerase Chain Reaction (PCR) and Sequencing

Total genomic DNA was extracted from one to six legs of the individual mosquitoes using Qiagen DNeasy kit (Qiagen) following manufacturer's instructions. The standard 658 bp *Cytochorme c oxidase subunit I* (*cox I*) gene was amplified using the universal primer pair LCO1490 (5′‐GGT CAA CAA ATC ATA AAG ATA TTG G‐3′) and HCO2198 (TAA ACT TCA GGG TGA CCA AAA AAT CA‐3′) (Folmer et al. 1994). Each amplification was performed in 25 μL reaction which included 3ul DNA template 12.5 μL Taq 2X Mastermix (NEB), 0.5 μL of forward and reverse primers (10 μM) and 8.5 μL of H_2_O. PCR parameters were 95°C for 5 min and 35 cycles of 95°C for 30 s, 48°C for 45 s and 68°C for 1 min followed by a final extension step of 68°C for 5 min. PCR products were run in 2% agarose gel stained with gel red (Fisher biotech) and visualised in Alliance Q9 imager (Uvitec).

PCR products showing positive clear bands were purified using ExoSAP‐IT (Thermo Fisher Scientific). Following the clean‐up of the PCR product, the ABI Big Dye Terminator v3.1 system was used for the sequencing reaction. Capillary electrophoresis was performed on the samples using 16‐capillary genetic analyser (ABI 3130 genetic analyser, Thermo Fisher Scientific). All sequences were uploaded to the Barcode of Life Data Systems (BOLD) and can be found under the project ‘Western Australian Mosquitoes’.

### 2.3 DNA Sequence Analysis

The bi‐directional trace files of the sequences were trimmed for low quality bases and edited using the Geneious Prime 2020.1.2 (https://www.geneious.com). The forward and reverse sequences were assembled using de novo assembly function in Geneious Prime. The sequences were aligned using MAFFT version 7.450 (Katoh et al. 2002; Katoh and Standley 2013) embedded in Geneious Prime. The consensus sequences along with metadata, were uploaded to the BOLD. The Refined Single Linkage (RESL) algorithm within BOLD was used to assign Barcode Index Numbers (BINs) to the COI dataset (Ratnasingham and Hebert 2013). The RESL algorithm uses a two‐step procedure: an initial clustering at a 2.2% divergence threshold followed by a refinement step using Markov clustering. It also allows a direct comparison of the COI dataset with all sequences present in the BOLD database.

The maximum likelihood (ML) phylogenetic analyses were performed based on the general time‐reversible model with GTR+G+I model using MEGA X software (Kumar et al. 2018) with bootstrapping for 1000 replicates to assess nodal support. All phylogenetic trees were visualised and edited using FigTree v1.4.4 (https://tree.bio.ed.ac.uk/software/Figtree/). To estimate intra‐ and interspecific genetic distances, Kimura 2 Parameter (K2P) (Kimura 1980) within MEGA X was used. The number of haplotypes, haplotype diversity, parsimony informative sites, variable sites and GC conten were estimated using DNA sequence polymorphism software (DnaSP, version 6) (Rozas et al. 2017). The presence and absence of the ‘barcode gap’ were evaluated using BOLD's barcode gap analysis function. For species delimitation, the assemble species by automatic partitioning (ASAP) method based on K2P distance and BIN‐RESL algorithm method were used.

## 3 Results

### 3.1 Morphological Identification

Of the 177 specimens, 91 were collected from the Kimberley region situated in the far north of WA while 86 were collected from Southern WA (Perth, Peel and South West regions). Information about the exact location of each specimen is presented in Table S1. The name of the species was verified using the Mosquito Taxonomic Inventory (Harbach 2023). The tribe Aedini, which includes approximately one quarter of known mosquito species and contains the genus *Aedes*, has undergone several taxonomic revisions however these classifications are not commonly used in Australia. To avoid confusion and for comparison with previous literature, both traditional and revised names according to the most recent revision (Reinert, Harbach, and Kitching 2009) are presented in Table 1. A total of 45 species from 9 genera were identified: *Aedeomyia* (1 species), *Aedes* (18 species), *Anopheles* (6 species), *Coquillettidia* (2 species), *Culex* (13 species), *Culiseta* (1 species), *Mansonia* (1 species), *Tripteroides* (2 species), and *Uranotaenia* (1 species).

**TABLE 1:** Species names of mosquitoes of the tribe Aedini used in this study and the new names according to recent revisions.

Species names used in this study	Name according to recent revisions
*Aedes notoscriptus* (Skuse, 1889)	*Rampamyia notoscripta*
*Aedes vigilax* (Skuse, 1889)	*Ochlerotatus vigilax*
*Aedes alboannulatus* (Macquart, 1850)	*Dobrotworskyius alboannulatus*
*Aedes camptorhynchus* (Thomson, 1869)	*Ochlerotatus camptorhynchus*
*Aedes ratcliffei* (Marks, 1959)	*Ochlerotatus ratcliffei*
*Aedes clelandi* (Taylor, 1914)	*Ochlerotatus clelandi*
*Aedes hesperonotius* (Marks, 1959)	*Ochlerotatus hesperonotius*
*Aedes nigrithorax* (Macquart, 1847)	*Ochlerotatus nigrithorax*
*Aedes turneri* (Marks, 1963)	*Ochlerotatus turneri*
*Aedes stricklandi* (Edwards, 1912)	*Ochlerotatus stricklandi*
*Aedes elchoensis* (Taylor, 1929)	*Macleaya elchoensis*
*Aedes tremulus* (Theobald, 1903)	*Macleaya tremula*
*Aedes daliensis* (Taylor, 1916)	*Ochlerotatus daliensis*
*Aedes normanensis* (Taylor, 1915)	*Ochlerotatus normanensis*
*Aedes lineatopennis* (Ludlow, 1905)	*Neomelaniconion lineatopenne*
*Aedes mallochi* (Taylor, 1944)	*Ochlerotatus mallochi*
*Aedes pecuniosus* (Edwards, 1922)	*Molpemyia pecuniosa*
*Aedes alternans* (Westwood, 1835)	*Mucidus alternans*
*Aedes albopictus* (Skuse, 1895)	*Stegomyia albopicta*

### 3.2 Analyses of COI Barcodes

The COI sequences of the mosquito specimens in this study were adenosine and thymine‐rich (AT‐rich), with an average nucleotide composition of adenine (A) = 29.2%, thymine (T) = 39.4%, guanine (G) = 15.3% and cytosine (C) = 16.1%. DNA polymorphism analyses of the 177 COI sequences showed 359 monomorphic invariable sites and 278 polymorphic variable sites with 244 parsimony informative and 34 singleton variable sites. Haplotype analysis revealed 134 haplotypes. The nucleotide alignments of each species were also used to calculate haplotype number of haplotypes and haplotype diversity and the results are presented in Table 2.

**TABLE 2:** List of mosquito species barcoded in this study, locations, BOLD Process IDs, number of haplotypes, polymorphic sites and haplotype diversity.

Species	Region	Town/City	BOLD Process IDs	*n*	*h*	Polymorphic sites	Haplotype diversity (±SD)
*Aedeomyia catasticta*	Kimberley	Wyndham	WAMOS056‐21 to WAMOS058‐21	3	3	6	1 ± 0.3
*Aedes alboannulatus*	South West	Mandurah	WAMOS102‐21 to WAMOS103‐21	2	4	13	0.9 ± 0.2
		Quindalup	WAMOS104‐21	1			
		Mandurah	WAMOS105‐21 to WAMOS106‐21	2			
*Ae. alternans*	Kimberley	Derby	WAMOS165‐21 to WAMOS166‐21	2	3	14	1 ± 0.3
		Wyndham	WAMOS167‐21	1			
*Ae. Camptorhynchus*	South West	Mandurah	WAMOS107‐21	1	2	3	0.4 ± 0.1
			WAMOS114‐21 to WAMOS118‐21	5			
		Australind	WAMOS108‐21	1			
		Eaton	WAMOS109‐21 to WAMOS110‐21	2			
		Capel	WAMOS111‐21	1			
		Stratham	WAMOS112‐21	1			
		Abbey	WAMOS113‐21	1			
*Ae. clelandi*	South West	Eaton	WAMOS122‐21	1	6	13	0.9 ± 0.1
		Mandurah	WAMOS123‐21	1			
			WAMOS126‐21 to WAMOS128‐21	3			
		Busselton	WAMOS124‐21	1			
		Quindalup	WAMOS125‐21	1			
*Ae. daliensis*	Kimberley	Derby	WAMOS144‐21 to WAMOS145‐21	2	2	1	1 ± 0.5
*Ae. elchoensis*	Kimberley	Wyndham	WAMOS140‐21	1	1	NC	NC
*Ae. hesperonotius*	Southwest	Busselton	WAMOS129‐21	1	2	5	1 ± 0.5
		Mandurah	WAMOS130‐21	1			
*Ae. lineatopennis*	Kimberley	Derby	WAMOS149‐21	1	3	2	0.7 ± 0.2
		Wyndham	WAMOS150‐21 to WAMOS153‐21	4			
*Ae. mallochi*	South West	Stratham	WAMOS154‐21	1	2	1	1 ± 0.5
		Capel	WAMOS155‐21	1			
*Ae. nigrithorax*	South West	Bunbury	WAMOS131‐21 to WAMOS132‐21	2	4	12	1 ± 0.2
		Mandurah	WAMOS133‐21 to WAMOS134‐21	2			
*Ae. normanensis*	Kimberley	Derby	WAMOS146‐21 to WAMOS148‐21	3	2	8	0.7 ± 0.3
*Ae. notoscriptus*	Kimberley	Broome	WAMOS083‐21 to WAMOS085‐21	3	4	21	0.8 ± 0.2
		Wyndham	WAMOS088‐21	1			
	South West	Eaton	WAMOS086‐21	1			
		Quindalup	WAMOS087‐21	1			
*Ae. pecuniosus*	Kimberley	Derby	WAMOS156‐21	1	3	2	1 ± 0.3
		Wyndham	WAMOS157‐21 to WAMOS158‐21	2			
*Ae. ratcliffei*	South West	Eaton	WAMOS119‐21	1	2	3	0.7 ± 0.3
		Busselton	WAMOS120‐21	1			
		Australind	WAMOS121‐21	1			
*Ae. stricklandi*	South West	Australind	WAMOS139‐21	1	1	NC	NC
*Ae. tremulus*	Kimberley	Broome	WAMOS141‐21 to WAMOS142‐21	2	3	17	1 ± 0.3
		Derby	WAMOS143‐21	1			
*Ae. turneri*	South West	Eaton	WAMOS135‐21	1	3	5	0.8 ± 0.2
		Busselton	WAMOS136‐21	1			
		Abbey	WAMOS137‐21	1			
		Mandurah	WAMOS138‐21	1			
*Ae. vigilax*	Kimberley	Broome	WAMOS089‐21 to WAMOS090‐21	2	8	13	0.8 ± 0.1
		Derby	WAMOS091‐21 to WAMOS092‐21	2			
		Wyndham	WAMOS100‐21 to WAMOS101‐21	2			
	South West	Mandurah	WAMOS093‐21	1			
			WAMOS096‐21 to WAMOS099‐21	4			
		Australind	WAMOS094‐21	1			
		Quindalup	WAMOS095‐21	1			
*Anopheles amictus*	Kimberley	Derby	WAMOS059‐21 to WAMOS060‐21	2	3	8	1 ± 0.3
		Wyndham	WAMOS061‐21	1			
*An. annulipes* sensu lato	Kimberley	Derby	WAMOS066‐21 to WAMOS067‐21	2	7	69	0.9 ± 0.1
		Wyndham	WAMOS075‐21	1			
	South West	Australind	WAMOS068‐21	1			
			WAMOS074‐21	1			
		Eaton	WAMOS069‐21	1			
		Quindalup	WAMOS070‐21	1			
		Mandurah	WAMOS071‐21 to WAMOS073‐21	3			
*An. atratipes*	South West	Eaton	WAMOS078‐21 to WAMOS079‐21	2	2	1	1 ± 0.5
*An. bancroftii*	Kimberley	Wyndham	WAMOS080‐21 to WAMOS082‐21	3	3	3	1 ± 0.3
*An. Hilli*	Kimberley	Wyndham	WAMOS062‐21 to WAMOS065‐21	4	4	4	1 ± 0.2
*An. meraukensis*	Kimberley	Wyndham	WAMOS076‐21 to WAMOS077‐21	2	2	1	1 ± 0.5
*Coquillettidia* species near *linealis*	South West	Australind	WAMOS168‐21 to WAMOS169‐21	2	3	6	1 ± 0.3
		Mandurah	WAMOS170‐21	1			
*Cq. xanthogaster*	Kimberley	Wyndham	WAMOS171‐21 to WAMOS173‐21	3	3	4	1 ± 0.3
*Culex palpalis*	Kimberley	Wyndham	WAMOS020‐21 to WAMOS023‐21	4	4	4	1 ± 0.2
*Cx. annulirostris*	Kimberley	Broome	WAMOS001‐21 to WAMOS008‐21	8	16	33	1 ± 0.0
		Derby	WAMOS009‐21 to WAMOS010‐21	2			
		Wyndham	WAMOS018‐21 to WAMOS019‐21	2			
	South West	Mandurah	WAMOS011‐21	1			
			WAMOS015‐21 to WAMOS017‐21	3			
		Australind	WAMOS012‐21	1			
		Eaton	WAMOS013‐21	1			
		Quindalup	WAMOS014‐21	1			
*Cx. australicus*	South West	Mandurah	WAMOS033‐21 to WAMOS034‐21	2	3	7	1 ± 0.3
		Australind	WAMOS035‐21	1			
*Cx. Bitaeniorhynchus*	Kimberley	Wyndham	WAMOS040‐21 to WAMOS042‐21	3	2	1	0.7 ± 0.3
*Cx. gelidus*	Kimberley	Wyndham	WAMOS049‐21 to WAMOS050‐21	2	1	0	0.0 ± 0.0
*Cx. globlocoxitus*	South West	Mandurah	WAMOS028‐21	1	4	9	0.9 ± 0.2
			WAMOS031‐21	1			
		Australind	WAMOS029‐21	1			
			WAMOS032‐21	1			
		Eaton	WAMOS030‐21	1			
*Cx. Hilli*	Kimberley	Wyndham	WAMOS051‐21	1	1	NC	NC
*Cx. Latus*	South West	Busselton	WAMOS052‐21	1	1	NC	NC
*Cx. pipiens* biotype *molestus*	Perth	Lesmurdie	WAMOS026‐21 to WAMOS027‐21	2	1	0	0.0 ± 0.0
*Cx. pullus*	Kimberley	Derby	WAMOS044‐21 to WAMOS045‐21	2	2	1	0.4 ± 0.2
		Wyndham	WAMOS046‐21 to WAMOS048‐21	3			
*Cx. Quinquefasciatus*	South West	Quindalup	WAMOS036‐21	1	4	7	1 ± 0.2
		Mandurah	WAMOS037‐21	1			
		Wonnerup	WAMOS038‐21	1			
		Eaton	WAMOS039‐21	1			
*Cx. sitiens*	Kimberley	Broome	WAMOS024‐21	1	2	2	1 ± 0.5
		Wyndham	WAMOS025‐21	1			
*Cx. starckeae*	Kimberley	Wyndham	WAMOS043‐21	1	1	NC	NC
*Culiseta atra*	South West	Eaton	WAMOS053‐21	1	2	3	1 ± 0.5
			WAMOS054‐21	1			
*Mansonia uniformis*	Kimberley	Wyndham	WAMOS174‐21 to WAMOS177‐21	4	3	2	0.8 ± 0.2
*Tripteroides atripes*	South West	Mandurah	WAMOS163‐21	1	2	1	1 ± 0.5
		Bunbury	WAMOS164‐21	1			
*Tp. punctolateralis*	Kimberley	Broome	WAMOS159‐21 to WAMOS161‐21	3	2	10	0.5 ± 0.3
		Derby	WAMOS162‐21	1			
*Uranotaenia albescens*	Kimberley	Wyndham	WAMOS055‐21	1	1	NC	NC

Abbreviations: *h* = number of haplotypes, *N* = number of COI sequences, NC = not calculated (i.e., represented by only one specimen), SD = standard deviation.

### 3.3 BOLD BIN Analysis

The RESL clustering approach applied to the COI marker assigned 47 BINs for 45 species sequenced in this study (Table 3). BIN analysis revealed that 28 species perfectly clustered into respective single BIN. Six species were split into multiple BINs, which include: *Ae. nigrithorax* (BOLD: AAE2446 and ACS6163), *Ae. normanensis* (BOLD: AEI6697 and AAV4152), *Ae. notoscriptus* (BOLD: AAG3835, ADM7085 and ADM7086), *Ae. tremulus* (BOLD: AEI4268 and AAZ2708), *An. annulipes* s.l (BOLD: AAB2268, AAF0630, ABZ0076 and ACE2888) and *Cx. annulirostris* (BOLD: AAG3833 and AEG7306).

**TABLE 3:** Barcode Index Number (BIN) results and minimum interspecific distance based on barcode gap analysis in the BOLD database.

Species	Barcode Index Number (BIN) results			Nearest species	Distance to the nearest neighbour
	BIN ID	Total members	Count in project		
*Ad. catasticta*	ACV8061	6	3	*Ur. albescens*	11.47
*Ae. alboannulatus*	ABX6893	15	5	*Ae. camptorhynchus*	7.51
*Ae. alternans*	AAG3831	18	3	*Ae. nigrithorax*	11.85
*Ae. camptorhynchus*	ACB5426	110	12	*Ae. ratcliffei*	5.71
*Ae. clelandi*	AEI2751	9	7	*Ae. hesperonotius*	0.46
*Ae. daliensis*	AEI7628	2	2	*Ae. Clelandi*	10.07
*Ae. elchoensis*	AEI5460	1	1	*Ae. Turneri*	9.55
*Ae. hesperonotius*	AEI2751	9	2	*Ae. clelandi*	0.46
*Ae. lineatopennis*	ADJ4564	6	5	*Ae. nigrithorax*	10.25
*Ae. mallochi*	ACS5624	11	2	*Ae. camptorhynchus*	9.72
*Ae. nigrithorax*	AAE2446	21	3	*Ae. clelandi*	6.18
	ACS6163	7	1		
*Ae. normanensis*	AEI6697	2	2	*Ae. nigrithorax*	10.44
	AAV4152	3	1		
*Ae. notoscriptus*	AAG3835	12	4	*Ae. nigrithorax*	9.55
	ADM7085	32	1		
	ADM7086	8	1		
*Ae. pecuniosus*	AEI5196	3	3	*Ae. camptorhynchus*	10.95
*Ae. ratcliffei*	AEI8195	3	3	*Ae. camptorhynchus*	5.71
*Ae. stricklandi*	AEI1635	5	1	*Ae. turneri*	2.01
*Ae. tremulus*	AEI4268	2	2	*Ae. nigrithorax*	10.07
	AAZ2708	4	1		
*Ae. turneri*	AEI1635	5	4	*Ae. stricklandi*	2.01
*Ae. vigilax*	AAC1707	250	13	*Ae. nigrithorax*	8.86
*An. amictus*	AAF0754	7	3	*An. Hilli*	5.23
*An. annulipes* s.l	AAB2268	41	7	*An. meraukensis*	9.31
	AAF0630	5	1		
	ABZ0076	10	1		
	ACE2888	20	1		
*An. Atratipes*	AEI9151	2	2	*An. meraukensis*	10.94
*An. Bancroftii*	ADZ1695	10	3	*An. meraukensis*	9.84
*An. Hilli*	AAD3119	11	4	*An. amictus*	5.23
*An. meraukensis*	AAE5341	6	2	*An. annulipes*	9.31
*Cq*. sp. near *linealis*	AEI9641	3	3	*Co. xanthogaster*	13.14
*Cq. xanthogaster*	AAF1119	108	3	*Ur. albescens*	13.14
*Cx. annulirostris*	AAG3833	232	16	*Cx. sitiens*	0.15
	AEG7306	43	3		
*Cx. australicus*	AEW0336	248	3	*Cx. quinquefasciatus*	0
*Cx. bitaeniorhynchus*	AAJ7281	108	3	*Cx. starckeae*	6.18
*Cx. gelidus*	AAC6669	163	2	*Cx. australicus*	7.85
*Cx. globocoxitus*	AEW0336	248	5	*Cx. quinquefasciatus*	0
*Cx. hilli*	AEI7284	1	1	*Cx. bitaeniorhynchus*	8.01
*Cx. latus*	ACV5066	2	1	*Cx. pipiens*	9.74
*Cx. palpalis*	AAG3833	232	4	*Cx. sitiens*	0
*Cx. pipiens*	AAA4751	5980	2	*Cx. australicus*	2.96
*Cx. pullus*	ACW1597	9	5	*Cx. australicus*	6.85
*Cx. quinquefasciatus*	AEW0336	248	4	*Cx. globocoxitus*	0
*Cx. sitiens*	AAG3833	232	2	*Cx. palpalis*	0
*Cx. starckeae*	AEI0300	1	1	*Cx. bitaeniorhynchus*	6.18
*Cs. Atra*	ACV7089	40	2	*Cx. Latus*	11.69
*Ma. uniformis*	AAB7892	25	4	*Ae. nigrithorax*	13.81
*Tp. atripes*	ACS4308	9	2	*Tr. punctolateralis*	0.77
*Tp. punctolateralis*	ACS4308	9	4	*Tr. atripes*	0.77
*Ur. albescens*	AAG3844	40	1	*Cx. gelidus*	10.42

Some of the species shared the same BIN. All the members within *Cx. australicus*, *Cx. globocoxitus* and *Cx. quinquefasciatus* shared BIN BOLD: AEW0336. Likewise, 16 members from *Cx. annulirostris* and all of *Cx. palpalis* and *Cx. sitiens* shared the same BIN BOLD: AAG3833. The other three members of *Cx. annulirostris* were under the BIN BOLD: AEG7306. Also, the species pairs including *Ae. clelandi* and *Ae. hesperonotius* (BOLD: AEI2571), *Ae. stricklandi* and *Ae. turneri* (BOLD: AEI1635) and *Tripteroides atripes* and *Tp. punctolateralis* (BOLD: ACS4308) shared the same BINs. A total of 12 unique BINs were identified.

### 3.4 Intraspecific and Interspecific Sequence Divergence

**FIGURE 2:**
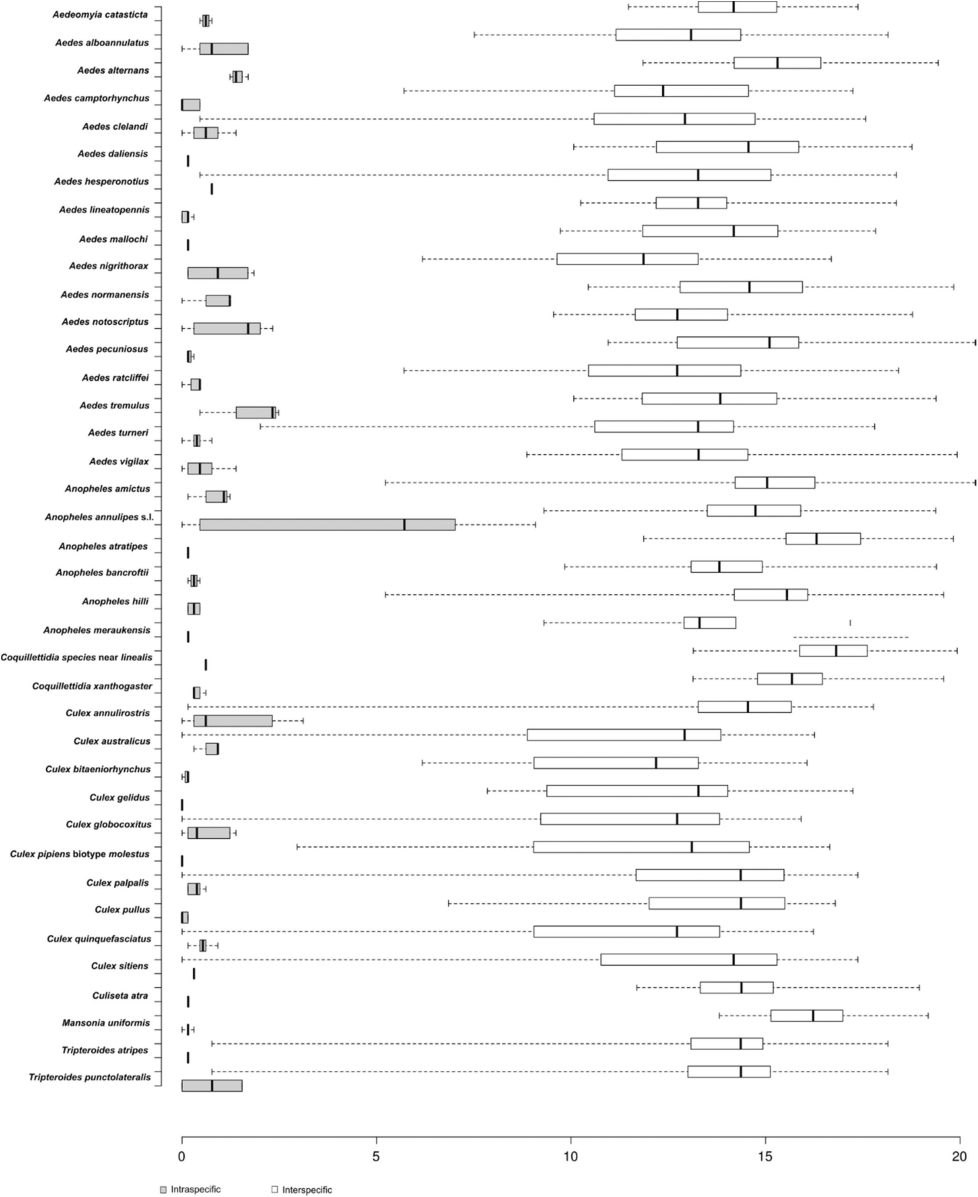
Intra and interspecies Kimura‐2 Parameter (K2P) genetic distances of COI sequences from WA mosquito species. Centre lines show medians; whiskers extend to the minimum and maximum values. Singleton species are excluded from the analysis.

The average intraspecific K2P distance of the 45 mosquito species was 1% (range 0%–9.1%). The maximum observed intraspecific divergence was 9.1% for *An. annulipes* s.l followed by 3.12 for *Cx. annulirostris* (Table 4). The average minimum interspecific genetic variation inferred by the distance to the nearest neighbour of the 45 mosquito species was 6.8% ranging from 0% to 13.8% (Table 3). The lowest minimum interspecific divergence of 0% was observed in the members of *Cx. pipiens* complex (*Cx. australicus*, *Cx. globocoxitus* and *Cx. quinquefasciatus*) and *Cx. sitiens* subgroup (*Cx. annulirostris*, *Cx. sitiens*, *Cx. palpalis*) followed by the species pairs *Ae. clelandi* and *Ae. hesperonotius* at 0.46% to each other. Another species pairs with low minimum interspecific divergence were *Tp. atripes* and *Tp. punctolateralis* (0.77%). These differences collectively account for all the overlap between intraspecific and interspecific differences seen in Figure 2. The highest minimum interspecific divergence was found in *Ma. uniformis* (closest to *Ae. nigrithorax*, 13.8%).

**TABLE 4:** The maximum observed intraspecific Kimura two‐parameter (K2P) distances among the COI sequences.

Species	Number of specimens	Average K2P distance (%)	Maximum observed K2P difference between intraspecific specimens (%)
*Aedeomyia catasticta*	3	0.61	0.77
*Aedes alboannulatus*	5	0.92	1.70
*Aedes alternans*	3	1.44	1.70
*Aedes lineatopennis*	5	0.12	0.30
*Aedes notoscriptus*	6	1.24	2.33
*Aedes pecuniosus*	3	0.20	0.30
*Aedes tremulus*	3	1.75	2.48
*Aedes camptorhynchus*	12	0.19	0.46
*Aedes clelandi*	7	0.66	1.39
*Aedes daliensis*	2	0.15	0.15
*Aedes hesperonotius*	2	0.77	0.77
*Aedes mallochi*	2	0.15	0.15
*Aedes nigrithorax*	4	0.95	1.85
*Aedes normanensis*	3	0.82	1.23
*Aedes ratcliffei*	3	0.30	0.46
*Aedes turneri*	4	0.38	0.77
*Aedes vigilax*	13	0.49	1.39
*Anopheles amictus*	3	0.82	1.23
*Anopheles annulipes* sensu lato	10	4.00	9.08
*Anopheles atratipes*	2	0.15	0.15
*Anopheles bancroftii*	3	0.31	0.46
*Anopheles hilli*	4	0.31	0.46
*Anopheles meraukensis*	2	0.16	0.16
*Coquillettidia* species near *linealis*	3	0.61	0.61
*Coquillettidia xanthogaster*	3	0.41	0.61
*Culex annulirostris*	19	1.07	3.12
*Culex australicus*	3	0.72	0.92
*Culex bitaeniorhynchus*	3	0.10	0.15
*Culex gelidus*	2	0.00	0.00
*Culex globlocoxitus*	5	0.61	1.38
*Culex pipiens* biotype *molestus*	2	0.00	0.00
*Culex palpalis*	4	0.36	0.61
*Culex pullus*	5	0.06	0.15
*Culex quinquefasciatus*	4	0.53	0.92
*Culex sitiens*	2	0.30	0.30
*Culiseta atra*	2	0.16	0.16
*Mansonia uniformis*	4	0.15	0.30
*Tripteroides atripes*	2	0.15	0.15
*Tripteroides punctolateralis*	4	0.77	1.54

### 3.5 Phylogenetic Analysis and Species Delimitation

**FIGURE 3:**
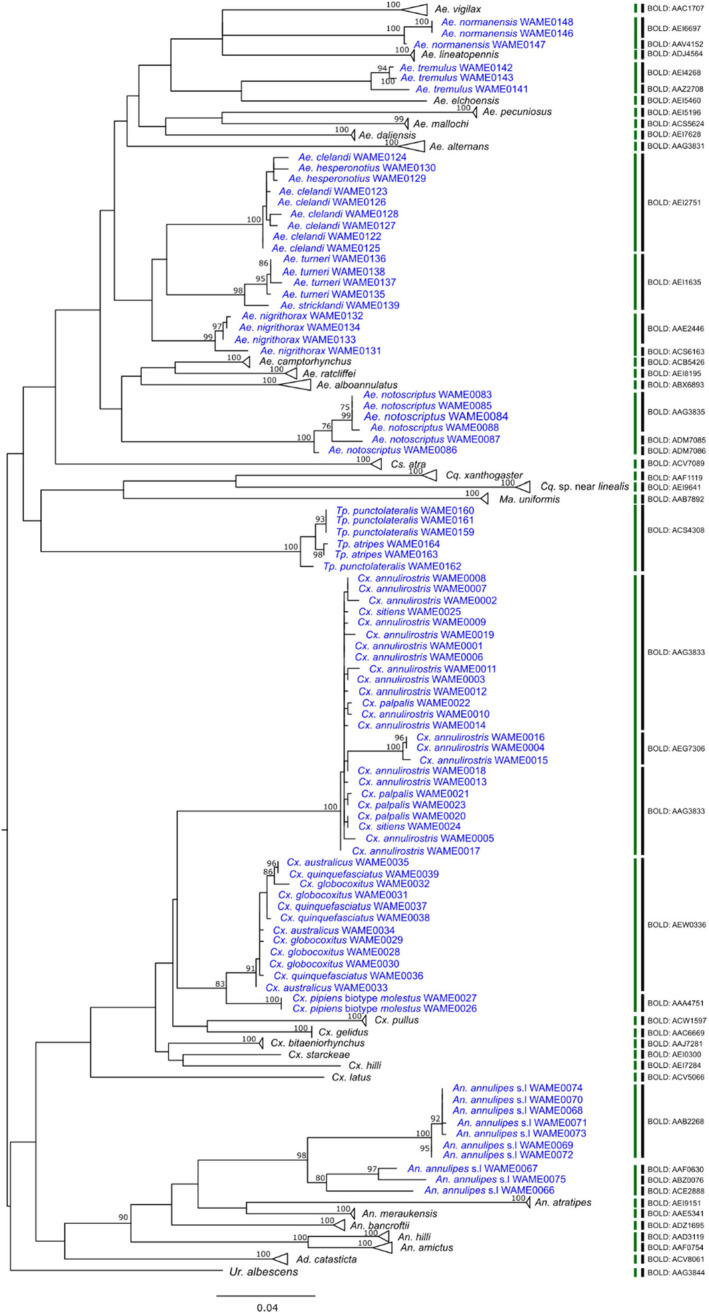
Maximum likelihood (ML) tree based on 177 cytochrome c oxidase subunit I (COI) sequences representing 45 mosquito species. Bootstrap support values are shown near each branch. Species are coloured blue if they share single BIN with other species or split into multiple BINs. Vertical bars indicate species delimited using BIN‐refined single linkage analysis (RESL) algorithm (black bar) and assemble species by automatic partitioning (ASAP) algorithm (green bar).

The maximum likelihood phylogenetic analysis revealed that most of the species demonstrated well‐supported clades (Figure 3). Three members of a *Cx. pipiens* subgroup, namely, *Cx. australicus*, *Cx. globocoxitus* and *Cx. quinquefasciatus* were clustered together. Similarly, *Cx. annulirostris*, *Cx. sitiens* and *Cx. palpalis* also formed a single clade with no separation between these species. They are part of a *Cx. sitiens* subgroup. A similar pattern was observed with species pairs *Tp. atripes* and *Tp. punctolateralis* and *Ae. clelandi* and *Ae. hesperonotius*. This clustering of these species' subgroups was, to a large extent, supported by the ASAP based on Kimura 2 parameter (K2P) distance and BIN‐RESL algorithm implemented in BOLD except that three *Cx. annulirostris* members were assigned a separate BIN (Table 3).

Both BOLD and ASAP failed to separate the *Ae. turneri* and *Ae. stricklandi* suggesting they are members of the same species subgroup. In contrast to the BIN‐RESL algorithm, ASAP worked well with *Ae. normanensis*, *Ae. nigrithorax*, *Ae. notoscriptus*, *Cx. annulirostris*, where members of respective species were kept together. ASAP method, however, failed to separate *An. hilli* and *An. amictus,* which BOLD assigned separate BINs. *An. annulipes* was split into four BINs by BOLD, while it was divided into two groups by ASAP.

## 4 Discussion

Accurate identification of mosquito species is crucial in selecting the optimal vector control approach for the target mosquito species (Sumruayphol et al. 2020) to successfully reduce mosquito populations. However, traditional morphological approaches to mosquito identification take years to develop the experience and knowledge for accurate identification of mosquito species and is a skill developed by few researchers across Australia. Mosquito identification is routinely performed by less experienced Local Government Environmental Health Officers taking considerable time and effort to correctly identify the mosquito fauna within their jurisdiction. Accurate identification is required to ensure targeted mosquito control options are deployed, considering the likely breeding sites and life history traits of the individual species identified that may be causing a nuisance or disease risk. The COI barcode of 177 mosquitoes, classified into 45 species and nine genera from the present study, provides support for DNA barcoding as a genetic approach for identifying mosquito species in WA. Furthermore, the addition of barcode data of 16 previously unbarcoded species (Table S2) has not only broadened the international reference library but also contributed to the Mosquito Barcoding Initiative (Linton 2009), further advancing the field of mosquito species identification and classification. As DNA barcoding libraries are further developed, efficiencies in mosquito identification through these techniques will provide more accurate assessments of mosquito populations and targeted approaches for their control.

The average AT‐richness of 68.6% in the DNA barcode sequences generated in the present study is consistent with similar studies describing AT‐richness in mosquito COI sequences (Wang et al. 2012; Chaiphongpachara et al. 2022). The majority of the barcoded species in this study formed distinct clusters in the phylogenetic analysis, thus confirming the utility of DNA barcoding.

This study reported ambiguous identification within the *Cx. sitiens* subgroup members (Lee, Bryan, and Russell 1989), namely, *Cx. annulirostris*, *Cx. sitiens* and *Cx. palpalis,* as their barcodes were all identified as *Cx. annulirostris*. These three members of *Cx. sitiens* subgroup are of significant interest in the Australasian region, owing to their wide geographical distribution and role in arbovirus transmission (Jansen et al. 2013). The difficulty in separating these species both morphologically and using non‐morphological methods is a known issue (Lee, Bryan, and Russell 1989; Chapman et al. 2000). Beebe et al. successfully employed PCR‐Restriction fragment length polymorphism (RFLP) in the ribosomal ITS1 sequences to discriminate between the three species (Beebe et al. 2002). Additionally, Hemmerter et al. reported COI identification of *Cx. annulirostris* and *Cx. palpalis* supporting the PCR‐RFLP method (Hemmerter, Slapeta, and Beebe 2009) but few samples from WA were included in that analysis. It is possible that in our present study we misidentified *Cx. annulirostris* as *Cx. palpalis* and/or *Cx. sitiens*. Nevertheless, a comprehensive investigation into the barcodes and morphological characteristics of the *Culex sitiens* subgroup members across a wide geographical range in Australia is warranted before drawing any definitive conclusions.

The *Culex pipiens* complex comprises four members in Australia: two indigenous, *Cx. australicus* and *Cx. globocoxitus* and two introduced, *Cx. quinquefasciatus* and *Cx. pipiens* biotype *molestus* (Russell 2012). The present study could not discriminate between the *Cx. australicus*, *Cx. globocoxitus* and *Cx. quinquefasciatus* while *Cx. pipiens* biotype *molestus* formed a separate cluster. The inability of the COI barcodes to distinguish the members of *Cx. pipiens* complex is known and the ACE‐2 marker has been used to differentiate between these species (Kasai et al. 2008; Laurito et al. 2013; Lee et al. 2012; Smith and Fonseca 2004). A similar Australian study from Victoria targeting COI barcodes reported very low divergence (0%–1%) between *Cx. australicus* and *Cx. globocoxitus* and no divergence between *Cx. quinquefasciatus* and *Cx. pipiens* biotype *molestus* (Batovska et al. 2016). It is now well established that the current COI barcoding method cannot discriminate between *Cx. pipiens* complex members; therefore, the use of other barcodes should be explored.

Other species groups with low genetic divergence found in this study were species pairs *Ae. clelandi* and *Ae. hesperonotius*; *Tp. atripes* and *Tp. punctolateralis*; and *Ae. turneri* and Ae. *stricklandi*. Among them *Ae. clelandi*, *Ae. hesperonotius*, *Tp. punctolateralis*, *Ae. turneri* and *Ae. stricklandi* were barcoded for the first time and are new additions to the BOLD and Genbank databases. It is to be noted that these species pairs are morphologically very similar (Liehne 1991; Webb and Russell 2016). Although COI identification of the former two species pairs could not be made, a phylogenetic tree assisted in discriminating between *Ae. turneri* and *Ae. stricklandi* in this study.

Species complexes and subgroups can confuse identification and create issues in the application of the ‘barcode gap’ (Candek and Kuntner 2015), the difference between intra‐ and interspecific genetic distances and is effective in species identification (Meier, Zhang, and Ali 2008; Sheraliev and Peng 2021). Several studies have shown the absence of a barcode gap in mosquitoes that are closely related or that belong to species complexes (Chaiphongpachara et al. 2022; Batovska et al. 2016; Meier, Zhang, and Ali 2008; Cywinska, Hunter, and Hebert 2006; Versteirt et al. 2015). Despite the lack of a barcode gap in some groups in this study, all other species clustered separately, thereby validating the diagnostic competence of DNA barcoding using COI. Additionally, phylogenetic analysis in concert with species delimitation methods can assist in correctly identifying species (Chaiphongpachara et al. 2022). Thus, phylogenetic analysis remains an essential aspect of DNA barcoding for species assessment.

*Anopheles annulipes* sensu lato, meaning *An. annulipes* in the broad sense, is a complex of genetically distinct but morphologically similar mosquitoes with at least 15 sibling species (Foley et al. 2007; Foley, Bryan, and Wilkerson 2007). A previous study by Foley et al. analysed the barcodes COI, COII, EF‐1α and ITS2 of *An. annulipes* s.l from across Australia found two major clades, one in northern Australia and one mainly in the south of the country (Foley et al. 2007). The COI analysis of 10 *An. annulipes* s.l species in the present study corroborated the earlier findings of Foley et al. Notably, we identified two well‐supported clades of *An. annulipes* s.l, with strong bootstrap support, one comprising three northern WA members and the other with seven members from southwest WA (Figure 3). Although ASAP analysis also split them into two groups, the BIN‐RESL algorithm further partitioned the three members from northern WA into three BINs, increasing the total number of BINs to four. Furthermore, the intraspecific divergence based on K2P distance was highest within *An. annulipes* s.l compared to other species. Therefore, this study reinforces the species richness of *An. annulipes* s.l and with further sampling, additional sibling species will likely be discovered.

Northern WA has a higher species diversity of *Anopheles* species compared to the south, with at least nine species extant (Liehne 1991). There was no publicly available COI data for *An. atratipes*, a single short (258 bp) sequence available for each of *An. meraukensis*, *An. hilli* and *An. amictus* and none for Australian *An. bancroftii* species (Genbank and BOLD, accessed 11/01/2023). Although the vector potential of most of the *Anopheles* species in WA remains largely unknown, *An. bancroftii* is the known vector of malaria and *Wuchereria bancrofti* in Papua New Guinea (PNG) (Beebe et al. 2001). The inclusion of COI sequences of *Anopheles* species from this study into the Genbank and BOLD databases will aid in investigating genetic variations of these mosquito vectors as more sequences are added in the future.

For species delimitation in this study's COI dataset, the ASAP method performed better than the BIN‐RESL method. In contrast to the BIN‐RESL approach, ASAP did not split *Ae. normanensis*, *Ae. tremulus*, *Ae. nigrithorax*, *Ae. notoscriptus* and failed to separate *An. amictus* and *An. hilli*. The ASAP method used in this study was based on the K2P substitution model that builds partitions from the barcode database using the threshold values to distinguish between intra‐ and interspecific divergence (Puillandre, Brouillet, and Achaz 2021). On the other hand, the BIN‐RESL algorithm is based on assigning Operational Taxonomic Units (OTUs) and putative species from the BOLD database using RESL (Ratnasingham and Hebert 2013). Our findings indicate, in line with other studies, that more than one species delimitation method must be used when delineating species as each method possesses its own distinct advantages (Puillandre, Brouillet, and Achaz 2021). One limitation of our study is the limited number of representative samples for each species. A larger sample size would offer a more accurate depiction of haplotype diversity and intraspecific divergence.

The DNA barcodes for WA mosquitoes presented in this study will assist public health officials to strengthen arbovirus surveillance programs targeted at the identified mosquitoes' life history traits to enhance the success of mosquito control efforts. Furthermore, accurate identification can assist in determining the risk posed by mosquitoes (whether they are a vector of human or animal diseases) further enhancing public health response measures and potentially reducing the incidence of disease. By incorporating DNA barcoding as an additional identification tool, it becomes possible to overcome challenges associated with species identification, especially when dealing with morphologically similar subgroups and species complexes. DNA barcoding is useful when the mosquito species are physically damaged or if the larval stages cannot be distinguished from each other. As the DNA barcodes are further developed, greater efficiencies can be achieved in this approach for mosquito identification. DNA barcoding can be integrated with next‐generation sequencing (NGS) technology, allowing the simultaneous processing of thousands of samples in a cost‐effective single run (Ji et al. 2013). A similar barcode library has been created in Victoria, an Australian state located on the eastern coast of the country (Batovska et al. 2016), demonstrating its utility in identifying mosquitoes from bulk samples using NGS. (Batovska et al. 2018). Internationally, countries including Canada, India, China, Belgium, Thailand, Saudi Arabia, Mexico and Singapore have established COI barcode databases for mosquito species in their respective regions (Wang et al. 2012; Chaiphongpachara et al. 2022; Cywinska, Hunter, and Hebert 2006; Versteirt et al. 2015; Chan et al. 2014; Chan‐Chable et al. 2019; Kumar et al. 2007; Noureldin et al. 2022). Notably, these studies have also utilised the universal COI barcode, making the data generated in the present study compatible for comparative analyses.

## 5 Conclusions

We have successfully barcoded 45 species of Western Australian mosquitoes sampled across a wide spatial range, significantly expanding the barcode data for WA mosquitoes. Most species exhibited adequate genetic diversity in their barcodes, enabling reliable species identification. However, we also report low genetic divergence between certain species pairs, including *Ae. clelandi* and *Ae. hesperonotius*; *Tp. atripes* and *Tp. punctolateralis*; and *Ae. turneri* and *Ae. stricklandi*, indicating that COI alone may not be sufficient for species discrimination. Other barcode regions should be explored for reliable identification of these species. Most importantly, the barcodes generated in this study and accompanying analysis can serve as valuable resources for mosquito surveillance programs, aiding in species identification, enhancing understanding of evolutionary relationships and identifying patterns of associations among different mosquito species. We have submitted all COI nucleotide sequences from mosquitoes analysed in this study to BOLD and Genbank databases to allow an analysis of genetic variations related to the geographical distribution of these mosquito species.

## Author Contributions

**Binit Lamichhane:** conceptualization (equal), data curation (equal), formal analysis (equal), investigation (equal), methodology (equal), writing – original draft (lead), writing – review and editing (equal). **Craig Brockway:** investigation (equal), resources (equal), writing – review and editing (equal). **Kimberly Evasco:** investigation (equal), resources (equal), writing – review and editing (equal). **Jay Nicholson:** investigation (equal), resources (equal), writing – review and editing (equal). **Peter J. Neville:** investigation (equal), resources (equal), writing – review and editing (equal). **John S. Mackenzie:** resources (equal), writing – review and editing (equal). **David Smith:** funding acquisition (equal), investigation (equal), project administration (equal), resources (equal), software (equal), writing – review and editing (equal). **Allison Imrie:** conceptualization (equal), funding acquisition (equal), project administration (equal), resources (equal), software (equal), supervision (lead), writing – review and editing (equal).

## Conflicts of Interest

The authors declare no conflicts of interest.

## Supporting information


**Table S1.** Details of mosquito species used in this study.

**Table S2.** Barcode records and medical importance of mosquito species sequenced in this study.

## Data Availability

All barcode sequences used in this study are uploaded into the BOLD Database under the project Western Australia Mosquitoes (WAMOS) with BOLD accession numbers from WAMOS001‐21 to WAMOS177‐21. The sequences were also stored on NCBI GenBank under the accession numbers PP145112 to PP145288. The photographs of mosquito species described in this study can be found at the following web address: https://www.health.wa.gov.au/~/media/Corp/Documents/Health‐for/Mosquitoes/PDF/Species‐Sheets‐for‐Mosquitoes‐in‐WA‐updated.pdf
