# Supplementary material for: DNA Barcoding for the Identification of Adult Mosquitoes (Diptera: Culicidae) in Western Australia
Source: Ecol Evol. 2024 Nov 8;14(11):e70493. doi: 10.1002/ece3.70493 (PMC11549376; doi:10.1002/ece3.70493)
Supplement: Supplementary file 1 — Table S1. Details of mosquito species used in this study. [file ECE3-14-e70493-s002.docx]

Table S1 Details of mosquito species used in this study.

| Name | Region | Town/City | Latitude | Longitude | BOLD Process ID | Genbank Accession Number |
| --- | --- | --- | --- | --- | --- | --- |
| *Culex annulirostris* | Kimberley | Broome | -17.948731 | 122.236006 | WAMOS001-21 | PP145112 |
| *Culex annulirostris* | Kimberley | Broome | -17.920246 | 122.245442 | WAMOS002-21 | PP145113 |
| *Culex annulirostris* | Kimberley | Broome | -17.922043 | 122.506818 | WAMOS003-21 | PP145114 |
| *Culex annulirostris* | Kimberley | Broome | -17.754840 | 122.204801 | WAMOS004-21 | PP145115 |
| *Culex annulirostris* | Kimberley | Broome | -17.720115 | 122.209799 | WAMOS005-21 | PP145116 |
| *Culex annulirostris* | Kimberley | Broome | -17.754335 | 122.205101 | WAMOS006-21 | PP145117 |
| *Culex annulirostris* | Kimberley | Broome | -17.975387 | 122.344608 | WAMOS007-21 | PP145118 |
| *Culex annulirostris* | Kimberley | Broome | -17.951629 | 122.245535 | WAMOS008-21 | PP145119 |
| *Culex annulirostris* | Kimberley | Derby | -18.187000 | 125.563428 | WAMOS009-21 | PP145120 |
| *Culex annulirostris* | Kimberley | Derby | -17.427572 | 123.734634 | WAMOS010-21 | PP145121 |
| *Culex annulirostris* | Peel | Mandurah | -32.525350 | 115.776510 | WAMOS011-21 | PP145122 |
| *Culex annulirostris* | South West | Australind | -33.305830 | 115.696940 | WAMOS012-21 | PP145123 |
| *Culex annulirostris* | South West | Eaton | -33.312390 | 115.716390 | WAMOS013-21 | PP145124 |
| *Culex annulirostris* | South West | Quindalup | -33.627860 | 115.125710 | WAMOS014-21 | PP145125 |
| *Culex annulirostris* | Peel | Mandurah | -32.575149 | 115.808613 | WAMOS015-21 | PP145126 |
| *Culex annulirostris* | Peel | Mandurah | -32.556990 | 115.757200 | WAMOS016-21 | PP145127 |
| *Culex annulirostris* | Peel | Mandurah | -32.777090 | 115.694090 | WAMOS017-21 | PP145128 |
| *Culex annulirostris* | Kimberley | Wyndham | -15.806902 | 128.749983 | WAMOS018-21 | PP145129 |
| *Culex annulirostris* | Kimberley | Wyndham | -15.811719 | 128.729800 | WAMOS019-21 | PP145130 |
| *Culex palpalis* | Kimberley | Wyndham | -15.681621 | 128.727209 | WAMOS020-21 | PP145131 |
| *Culex palpalis* | Kimberley | Wyndham | -15.629404 | 128.729506 | WAMOS021-21 | PP145132 |
| *Culex palpalis* | Kimberley | Wyndham | -15.823151 | 128.731978 | WAMOS022-21 | PP145133 |
| *Culex palpalis* | Kimberley | Wyndham | -15.506779 | 128.834552 | WAMOS023-21 | PP145134 |
| *Culex sitiens* | Kimberley | Broome | -17.824502 | 122.215176 | WAMOS024-21 | PP145135 |
| *Culex sitiens* | Kimberley | Wyndham | -15.731263 | 128.699617 | WAMOS025-21 | PP145136 |
| *Culex pipiens* biotype *molestus* | Perth Metropolitian | Lesmurdie | -31.993439 | 116.056041 | WAMOS026-21 | PP145137 |
| *Culex pipiens* biotype *molestus* | Perth Metropolitian | Lesmurdie | -31.993439 | 116.056041 | WAMOS027-21 | PP145138 |
| *Culex globlocoxitus* | Peel | Mandurah | -32.525350 | 115.776510 | WAMOS028-21 | PP145139 |
| *Culex globlocoxitus* | South West | Australind | -33.240910 | 115.717160 | WAMOS029-21 | PP145140 |
| *Culex globlocoxitus* | South West | Eaton | -33.302121 | 115.723750 | WAMOS030-21 | PP145141 |
| *Culex globlocoxitus* | Peel | Mandurah | -32.575149 | 115.808613 | WAMOS031-21 | PP145142 |
| *Culex globlocoxitus* | South West | Australind | -33.200240 | 115.693710 | WAMOS032-21 | PP145143 |
| *Culex australicus* | Peel | Mandurah | -32.525350 | 115.776510 | WAMOS033-21 | PP145144 |
| *Culex australicus* | Peel | Mandurah | -32.575149 | 115.808613 | WAMOS034-21 | PP145145 |
| *Culex australicus* | South West | Australind | -33.305830 | 115.696940 | WAMOS035-21 | PP145146 |
| *Culex quinquefasciatus* | South West | Quindalup | -33.627860 | 115.125710 | WAMOS036-21 | PP145147 |
| *Culex quinquefasciatus* | Peel | Mandurah | -32.537950 | 115.771670 | WAMOS037-21 | PP145148 |
| *Culex quinquefasciatus* | South West | Wonnerup | -33.626944 | 115.401758 | WAMOS038-21 | PP145149 |
| *Culex quinquefasciatus* | South West | Eaton | -33.312390 | 115.716390 | WAMOS039-21 | PP145150 |
| *Culex bitaeniorhynchus* | Kimberley | Wyndham | -15.681621 | 128.727209 | WAMOS040-21 | PP145151 |
| *Culex bitaeniorhynchus* | Kimberley | Wyndham | -15.588415 | 128.247977 | WAMOS041-21 | PP145152 |
| *Culex bitaeniorhynchus* | Kimberley | Wyndham | -15.551447 | 128.258484 | WAMOS042-21 | PP145153 |
| *Culex starckeae* | Kimberley | Wyndham | -15.701501 | 128.698488 | WAMOS043-21 | PP145154 |
| *Culex pullus* | Kimberley | Derby | -17.731703 | 123.650222 | WAMOS044-21 | PP145155 |
| *Culex pullus* | Kimberley | Derby | -17.740388 | 123.610187 | WAMOS045-21 | PP145156 |
| *Culex pullus* | Kimberley | Wyndham | -15.603123 | 128.764790 | WAMOS046-21 | PP145157 |
| *Culex pullus* | Kimberley | Wyndham | -15.701501 | 128.698488 | WAMOS047-21 | PP145158 |
| *Culex pullus* | Kimberley | Wyndham | -15.583011 | 128.626134 | WAMOS048-21 | PP145159 |
| *Culex gelidus* | Kimberley | Wyndham | -15.796344 | 128.689465 | WAMOS049-21 | PP145160 |
| *Culex gelidus* | Kimberley | Wyndham | -15.577787 | 128.787299 | WAMOS050-21 | PP145161 |
| *Culex hilli* | Kimberley | Wyndham | -15.731263 | 128.699617 | WAMOS051-21 | PP145162 |
| *Culex latus* | South West | Busselton | -33.656089 | 115.342097 | WAMOS052-21 | PP145163 |
| *Culiseta atra* | South West | Eaton | -33.302121 | 115.723750 | WAMOS053-21 | PP145164 |
| *Culiseta atra* | South West | Eaton | -33.302121 | 115.723750 | WAMOS054-21 | PP145165 |
| *Uranotaenia albescens* | Kimberley | Wyndham | -15.782295 | 128.743978 | WAMOS055-21 | PP145166 |
| *Aedeomyia catasticta* | Kimberley | Wyndham | -15.629404 | 128.729506 | WAMOS056-21 | PP145167 |
| *Aedeomyia catasticta* | Kimberley | Wyndham | -15.885523 | 128.740379 | WAMOS057-21 | PP145168 |
| *Aedeomyia catasticta* | Kimberley | Wyndham | -15.537337 | 128.822952 | WAMOS058-21 | PP145169 |
| *Anopheles amictus* | Kimberley | Derby | -17.356173 | 123.737129 | WAMOS059-21 | PP145170 |
| *Anopheles amictus* | Kimberley | Derby | -17.739217 | 123.574147 | WAMOS060-21 | PP145171 |
| *Anopheles amictus* | Kimberley | Wyndham | -15.731263 | 128.699617 | WAMOS061-21 | PP145172 |
| *Anopheles hilli* | Kimberley | Wyndham | -15.588415 | 128.247977 | WAMOS062-21 | PP145173 |
| *Anopheles hilli* | Kimberley | Wyndham | -15.549131 | 128.259411 | WAMOS063-21 | PP145174 |
| *Anopheles hilli* | Kimberley | Wyndham | -15.496650 | 128.143190 | WAMOS064-21 | PP145175 |
| *Anopheles hilli* | Kimberley | Wyndham | -15.551447 | 128.258484 | WAMOS065-21 | PP145176 |
| *Anopheles annulipes sensu lato* | Kimberley | Derby | -17.675101 | 123.381074 | WAMOS066-21 | PP145177 |
| *Anopheles annulipes sensu lato* | Kimberley | Derby | -18.215540 | 125.578762 | WAMOS067-21 | PP145178 |
| *Anopheles annulipes sensu lato* | South West | Australind | -33.240910 | 115.717160 | WAMOS068-21 | PP145179 |
| *Anopheles annulipes sensu lato* | South West | Eaton | -33.302121 | 115.723750 | WAMOS069-21 | PP145180 |
| *Anopheles annulipes sensu lato* | South West | Quindalup | -33.627860 | 115.125710 | WAMOS070-21 | PP145181 |
| *Anopheles annulipes sensu lato* | Peel | Mandurah | -32.556990 | 115.757200 | WAMOS071-21 | PP145182 |
| *Anopheles annulipes sensu lato* | Peel | Mandurah | -32.574384 | 115.671806 | WAMOS072-21 | PP145183 |
| *Anopheles annulipes sensu lato* | Peel | Mandurah | -32.777090 | 115.694090 | WAMOS073-21 | PP145184 |
| *Anopheles annulipes sensu lato* | South West | Australind | -33.200240 | 115.693710 | WAMOS074-21 | PP145185 |
| *Anopheles annulipes sensu lato* | Kimberley | Wyndham | -15.811719 | 128.729800 | WAMOS075-21 | PP145186 |
| *Anopheles meraukensis* | Kimberley | Wyndham | -15.588415 | 128.247977 | WAMOS076-21 | PP145187 |
| *Anopheles meraukensis* | Kimberley | Wyndham | -15.434055 | 128.963180 | WAMOS077-21 | PP145188 |
| *Anopheles atratipes* | South West | Eaton | -33.312390 | 115.716390 | WAMOS078-21 | PP145189 |
| *Anopheles atratipes* | South West | Eaton | -33.302121 | 115.723750 | WAMOS079-21 | PP145190 |
| *Anopheles bancroftii* | Kimberley | Wyndham | -15.779323 | 128.729519 | WAMOS080-21 | PP145191 |
| *Anopheles bancroftii* | Kimberley | Wyndham | -15.791645 | 128.715299 | WAMOS081-21 | PP145192 |
| *Anopheles bancroftii* | Kimberley | Wyndham | -15.788356 | 128.683525 | WAMOS082-21 | PP145193 |
| *Aedes notoscriptus* | Kimberley | Broome | -17.948731 | 122.236006 | WAMOS083-21 | PP145194 |
| *Aedes notoscriptus* | Kimberley | Broome | -17.923487 | 122.218286 | WAMOS084-21 | PP145195 |
| *Aedes notoscriptus* | Kimberley | Broome | -17.958185 | 122.208454 | WAMOS085-21 | PP145196 |
| *Aedes notoscriptus* | South West | Eaton | -33.312390 | 115.716390 | WAMOS086-21 | PP145197 |
| *Aedes notoscriptus* | South West | Quindalup | -33.627860 | 115.125710 | WAMOS087-21 | PP145198 |
| *Aedes notoscriptus* | Kimberley | Wyndham | -15.779323 | 128.729519 | WAMOS088-21 | PP145199 |
| *Aedes vigilax* | Kimberley | Broome | -17.992564 | 122.204978 | WAMOS089-21 | PP145200 |
| *Aedes vigilax* | Kimberley | Broome | -17.720115 | 122.209799 | WAMOS090-21 | PP145201 |
| *Aedes vigilax* | Kimberley | Derby | -17.310760 | 123.630560 | WAMOS091-21 | PP145202 |
| *Aedes vigilax* | Kimberley | Derby | -17.427572 | 123.734634 | WAMOS092-21 | PP145203 |
| *Aedes vigilax* | Peel | Mandurah | -32.525350 | 115.776510 | WAMOS093-21 | PP145204 |
| *Aedes vigilax* | South West | Australind | -33.240910 | 115.717160 | WAMOS094-21 | PP145205 |
| *Aedes vigilax* | South West | Quindalup | -33.627860 | 115.125710 | WAMOS095-21 | PP145206 |
| *Aedes vigilax* | Peel | Mandurah | -32.489801 | 115.769933 | WAMOS096-21 | PP145207 |
| *Aedes vigilax* | Peel | Mandurah | -32.556990 | 115.757200 | WAMOS097-21 | PP145208 |
| *Aedes vigilax* | Peel | Mandurah | -32.777090 | 115.694090 | WAMOS098-21 | PP145209 |
| *Aedes vigilax* | Peel | Mandurah | -33.200240 | 115.693710 | WAMOS099-21 | PP145210 |
| *Aedes vigilax* | Kimberley | Wyndham | -15.603123 | 128.764790 | WAMOS100-21 | PP145211 |
| *Aedes vigilax* | Kimberley | Wyndham | -15.779323 | 128.729519 | WAMOS101-21 | PP145212 |
| *Aedes alboannulatus* | Peel | Mandurah | -32.525350 | 115.776510 | WAMOS102-21 | PP145213 |
| *Aedes alboannulatus* | Peel | Mandurah | -32.537950 | 115.771670 | WAMOS103-21 | PP145214 |
| *Aedes alboannulatus* | South West | Quindalup | -33.627860 | 115.125710 | WAMOS104-21 | PP145215 |
| *Aedes alboannulatus* | Peel | Mandurah | -32.574384 | 115.671806 | WAMOS105-21 | PP145216 |
| *Aedes alboannulatus* | Peel | Mandurah | -32.777090 | 115.694090 | WAMOS106-21 | PP145217 |
| *Aedes camptorhynchus* | Peel | Mandurah | -32.525350 | 115.776510 | WAMOS107-21 | PP145218 |
| *Aedes camptorhynchus* | South West | Australind | -33.240910 | 115.717160 | WAMOS108-21 | PP145219 |
| *Aedes camptorhynchus* | South West | Eaton | -33.312390 | 115.716390 | WAMOS109-21 | PP145220 |
| *Aedes camptorhynchus* | South West | Eaton | -33.302121 | 115.723750 | WAMOS110-21 | PP145221 |
| *Aedes camptorhynchus* | South West | Capel | -33.430610 | 115.622100 | WAMOS111-21 | PP145222 |
| *Aedes camptorhynchus* | South West | Stratham | -33.668355 | 115.267792 | WAMOS112-21 | PP145223 |
| *Aedes camptorhynchus* | South West | Abbey | -33.668355 | 115.267792 | WAMOS113-21 | PP145224 |
| *Aedes camptorhynchus* | Peel | Mandurah | -32.574384 | 115.671806 | WAMOS114-21 | PP145225 |
| *Aedes camptorhynchus* | Peel | Mandurah | -32.642500 | 115.646300 | WAMOS115-21 | PP145226 |
| *Aedes camptorhynchus* | Peel | Mandurah | -32.642500 | 115.646300 | WAMOS116-21 | PP145227 |
| *Aedes camptorhynchus* | Peel | Mandurah | -32.777090 | 115.694090 | WAMOS117-21 | PP145228 |
| *Aedes camptorhynchus* | Peel | Mandurah | -32.777090 | 115.694090 | WAMOS118-21 | PP145229 |
| *Aedes ratcliffei* | South West | Eaton | -33.302121 | 115.723750 | WAMOS119-21 | PP145230 |
| *Aedes ratcliffei* | South West | Busselton | -33.656089 | 115.342097 | WAMOS120-21 | PP145231 |
| *Aedes ratcliffei* | South West | Australind | -33.200240 | 115.693710 | WAMOS121-21 | PP145232 |
| *Aedes clelandi* | South West | Eaton | -33.302121 | 115.723750 | WAMOS122-21 | PP145233 |
| *Aedes clelandi* | Peel | Mandurah | -32.537950 | 115.771670 | WAMOS123-21 | PP145234 |
| *Aedes clelandi* | South West | Busselton | -33.656089 | 115.342097 | WAMOS124-21 | PP145235 |
| *Aedes clelandi* | South West | Quindalup | -33.627860 | 115.125710 | WAMOS125-21 | PP145236 |
| *Aedes clelandi* | Peel | Mandurah | -32.575149 | 115.808613 | WAMOS126-21 | PP145237 |
| *Aedes clelandi* | Peel | Mandurah | -32.574384 | 115.671806 | WAMOS127-21 | PP145238 |
| *Aedes clelandi* | Peel | Mandurah | -32.777090 | 115.694090 | WAMOS128-21 | PP145239 |
| *Aedes hesperonotius* | South West | Busselton | -33.656089 | 115.342097 | WAMOS129-21 | PP145240 |
| *Aedes hesperonotius* | Peel | Mandurah | -32.575149 | 115.808613 | WAMOS130-21 | PP145241 |
| *Aedes nigrithorax* | South West | Bunbury | -33.377250 | 115.636420 | WAMOS131-21 | PP145242 |
| *Aedes nigrithorax* | South West | Bunbury | -33.377250 | 115.636420 | WAMOS132-21 | PP145243 |
| *Aedes nigrithorax* | Peel | Mandurah | -32.575149 | 115.808613 | WAMOS133-21 | PP145244 |
| *Aedes nigrithorax* | Peel | Mandurah | -32.556990 | 115.757200 | WAMOS134-21 | PP145245 |
| *Aedes turneri* | South West | Eaton | -33.302121 | 115.723750 | WAMOS135-21 | PP145246 |
| *Aedes turneri* | South West | Busselton | -33.656089 | 115.342097 | WAMOS136-21 | PP145247 |
| *Aedes turneri* | South West | Abbey | -33.668355 | 115.267792 | WAMOS137-21 | PP145248 |
| *Aedes turneri* | Peel | Mandurah | -32.575149 | 115.808613 | WAMOS138-21 | PP145249 |
| *Aedes stricklandi* | South West | Australind | -33.200240 | 115.693710 | WAMOS139-21 | PP145250 |
| *Aedes elchoensis* | Kimberley | Wyndham | -15.788356 | 128.683525 | WAMOS140-21 | PP145251 |
| *Aedes tremulus* | Kimberley | Broome | -17.948731 | 122.236006 | WAMOS141-21 | PP145252 |
| *Aedes tremulus* | Kimberley | Broome | -17.923487 | 122.218286 | WAMOS142-21 | PP145253 |
| *Aedes tremulus* | Kimberley | Derby | -17.300591 | 123.626011 | WAMOS143-21 | PP145254 |
| *Aedes daliensis* | Kimberley | Derby | -17.294700 | 123.608400 | WAMOS144-21 | PP145255 |
| *Aedes daliensis* | Kimberley | Derby | -17.300591 | 123.626011 | WAMOS145-21 | PP145256 |
| *Aedes normanensis* | Kimberley | Derby | -17.740388 | 123.610187 | WAMOS146-21 | PP145257 |
| *Aedes normanensis* | Kimberley | Derby | -18.232622 | 125.587689 | WAMOS147-21 | PP145258 |
| *Aedes normanensis* | Kimberley | Derby | -17.427572 | 123.734634 | WAMOS148-21 | PP145259 |
| *Aedes lineatopennis* | Kimberley | Derby | -17.740388 | 123.610187 | WAMOS149-21 | PP145260 |
| *Aedes lineatopennis* | Kimberley | Wyndham | -15.796344 | 128.689465 | WAMOS150-21 | PP145261 |
| *Aedes lineatopennis* | Kimberley | Wyndham | -15.434055 | 128.963180 | WAMOS151-21 | PP145262 |
| *Aedes lineatopennis* | Kimberley | Wyndham | -15.506779 | 128.834552 | WAMOS152-21 | PP145263 |
| *Aedes lineatopennis* | Kimberley | Wyndham | -15.564874 | 128.202557 | WAMOS153-21 | PP145264 |
| *Aedes mallochi* | South West | Stratham | -33.470412 | 115.568240 | WAMOS154-21 | PP145265 |
| *Aedes mallochi* | South West | Capel | -33.550500 | 115.514730 | WAMOS155-21 | PP145266 |
| *Aedes pecuniosus* | Kimberley | Derby | -17.427572 | 123.734634 | WAMOS156-21 | PP145267 |
| *Aedes pecuniosus* | Kimberley | Wyndham | -15.799493 | 128.763827 | WAMOS157-21 | PP145268 |
| *Aedes pecuniosus* | Kimberley | Wyndham | -15.577787 | 128.787299 | WAMOS158-21 | PP145269 |
| *Tripteroides punctolateralis* | Kimberley | Broome | -17.824502 | 122.215176 | WAMOS159-21 | PP145270 |
| *Tripteroides punctolateralis* | Kimberley | Broome | -17.992564 | 122.204978 | WAMOS160-21 | PP145271 |
| *Tripteroides punctolateralis* | Kimberley | Broome | -17.967289 | 122.222634 | WAMOS161-21 | PP145272 |
| *Tripteroides punctolateralis* | Kimberley | Derby | -17.731703 | 123.650222 | WAMOS162-21 | PP145273 |
| *Tripteroides atripes* | Peel | Mandurah | -32.525350 | 115.776510 | WAMOS163-21 | PP145274 |
| *Tripteroides atripes* | South West | Bunbury | -33.377250 | 115.636420 | WAMOS164-21 | PP145275 |
| *Aedes alternans* | Kimberley | Derby | -17.364928 | 123.667161 | WAMOS165-21 | PP145276 |
| *Aedes alternans* | Kimberley | Derby | -17.351157 | 123.669538 | WAMOS166-21 | PP145277 |
| *Aedes alternans* | Kimberley | Wyndham | -15.806902 | 128.749983 | WAMOS167-21 | PP145278 |
| *Coquillettidia species near linealis* | South West | Australind | -33.240910 | 115.717160 | WAMOS168-21 | PP145279 |
| *Coquillettidia species near linealis* | South West | Australind | -33.305830 | 115.696940 | WAMOS169-21 | PP145280 |
| *Coquillettidia species near linealis* | Peel | Mandurah | -32.575149 | 115.808613 | WAMOS170-21 | PP145281 |
| *Coquillettidia xanthogaster* | Kimberley | Wyndham | -15.796344 | 128.689465 | WAMOS171-21 | PP145282 |
| *Coquillettidia xanthogaster* | Kimberley | Wyndham | -15.597582 | 128.279243 | WAMOS172-21 | PP145283 |
| *Coquillettidia xanthogaster* | Kimberley | Wyndham | -15.714291 | 128.255789 | WAMOS173-21 | PP145284 |
| *Mansonia uniformis* | Kimberley | Wyndham | -15.629404 | 128.729506 | WAMOS174-21 | PP145285 |
| *Mansonia uniformis* | Kimberley | Wyndham | -15.731263 | 128.699617 | WAMOS175-21 | PP145286 |
| *Mansonia uniformis* | Kimberley | Wyndham | -15.788872 | 128.704432 | WAMOS176-21 | PP145287 |
| *Mansonia uniformis* | Kimberley | Wyndham | -15.796344 | 128.689465 | WAMOS177-21 | PP145288 |
