# Supplementary material for: DNA Barcoding for the Identification of Adult Mosquitoes (Diptera: Culicidae) in Western Australia
Source: Ecol Evol. 2024 Nov 8;14(11):e70493. doi: 10.1002/ece3.70493 (PMC11549376; doi:10.1002/ece3.70493)
Supplement: Supplementary file 2 — Table S2. Barcode records and medical importance of mosquito species sequenced in this study. [file ECE3-14-e70493-s001.docx]

Table S2: Barcode records and medical importance of mosquito species sequenced in this study

| **Species** | **Barcode Sequence Availability in BOLD Database prior to this study (As of 11/01/2024)** | **Medical Importance** |
| --- | --- | --- |
| *Culex annulirostris* | Available | Yes |
| *Culex palpalis* | Available | Unknown |
| *Culex sitiens* | Available | Unknown |
| *Culex pipiens biotype molestus* | Available | Yes |
| *Culex globlocoxitus* | Available | Unknown |
| *Culex australicus* | Available | Unknown |
| *Culex quinquefasciatus* | Available | Yes |
| *Culex bitaeniorhynchus* | Available | Unknown |
| *Culex starckeae* | New record | Unknown |
| *Culex pullus* | New record | Unknown |
| *Culex gelidus* | Available | Unknown |
| *Culex hilli* | New record | Unknown |
| *Culex latus* | New record | Unknown |
| *Culiseta atra* | New record | Unknown |
| *Uranotaenia albescens* | Available | Unknown |
| *Aedeomyia catasticta* | No Australian sequences available | Unknown |
| *Anopheles amictus* | No public record | Unknown |
| *Anopheles hilli* | No public record | Unknown |
| *Anopheles annulipes* sensu lato | Available | Yes |
| *Anopheles meraukensis* | No public record | Unknown |
| *Anopheles atratipes* | New record | Unknown |
| *Anopheles bancroftii* | No Australian sequences available | Yes |
| *Aedes notoscriptus* | Available | Yes |
| *Aedes vigilax* | Available | Yes |
| *Aedes alboannulatus* | Available | Yes |
| *Aedes camptorhynchus* | Available | Yes |
| *Aedes ratcliffei* | New record | Unknown |
| *Aedes clelandi* | New record | Unknown |
| *Aedes hesperonotius* | New record | Unknown |
| *Aedes nigrithorax* | New record | Unknown |
| *Aedes turneri* | New record | Unknown |
| *Aedes stricklandi* | New record | Unknown |
| *Aedes elchoensis* | New record | Unknown |
| *Aedes tremulus* | Available | Unknown |
| *Aedes daliensis* | New record | Unknown |
| *Aedes normanensis* | Available | Unknown |
| *Aedes lineatopennis* | Available | Unknown |
| *Aedes mallochi* | Available | Unknown |
| *Aedes pecuniosus* | New record | Unknown |
| *Tripteroides punctolateralis* | New record | Unknown |
| *Tripteroides atripes* | Available | Unknown |
| *Aedes alternans* | Available | Unknown |
| *Coquillettidia* species near *linealis* | Available | Yes |
| *Coquillettidia xanthogaster* | Available | Unknown |
| *Mansonia uniformis* | Available | Unknown |
